# Tris(2,2′-bipyridine-κ^2^ *N*:*N*′)cobalt(III) trichloride tetra­hydrate

**DOI:** 10.1107/S1600536808038154

**Published:** 2008-11-22

**Authors:** Wen Liu, Wei Xu, Jian-Li Lin, Hong-Zhen Xie

**Affiliations:** a State Key Laboratory Base of Novel Functional Materials and Preparation Science, Faculty of Materials Science and Chemical Engineering, Ningbo University, Ningbo 315211, People’s Republic of China

## Abstract

The title compound, [Co(C_10_H_8_N_2_)_3_]Cl_3_·4H_2_O, contains discrete [Co(bpy)_3_]^3+^ cations (bpy is 2,2′-bipyridine), Cl^−^ anions and water mol­ecules. The [Co(bpy)_3_]^3+^ complex cation exhibits *C*_2_ symmetry with the twofold axis through the central Co atom and bis­ecting one bpy ligand and one of the Cl^−^ anions. The four solvent water mol­ecules and the remaining two Cl^−^ anions lie on a mirror plane. Hydrogen-bond inter­actions define a two-dimensional layer structure parallel to (100), which consists of seven-membered [Cl_2_(H_2_O)_5_], eight-membered [Cl_4_(H_2_O)_4_] and ten-membered [Cl_2_(H_2_O)_8_] rings.

## Related literature

For general background, see: Liu *et al.* (1996 ▶); Nauta & Miller (2000 ▶); Ludwig (2001 ▶); Saha & Bernal (2005 ▶); Reger *et al.* (2006 ▶); Li *et al.* (2007 ▶); Mir & Vittal (2007 ▶). For related structures, see: Hernández-Molina *et al.* (1998 ▶).
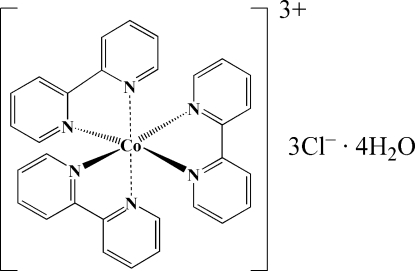


## Experimental

### 

#### Crystal data

[Co(C_10_H_8_N_2_)_3_]Cl_3_·4H_2_O
*M* *_r_* = 705.90
Orthorhombic, 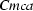

*a* = 20.171 (4) Å
*b* = 23.170 (5) Å
*c* = 13.316 (3) Å
*V* = 6223 (2) Å^3^
*Z* = 8
Mo *K*α radiation
μ = 0.86 mm^−1^
*T* = 295 (2) K
0.12 × 0.10 × 0.08 mm

#### Data collection

Bruker P4 diffractometer
Absorption correction: ψ scan (*XSCANS*; Siemens, 1996 ▶) *T*_min_ = 0.905, *T*_max_ = 0.929
3430 measured reflections
2824 independent reflections
1980 reflections with *I* > 2σ(*I*)
*R*_int_ = 0.049
3 standard reflections every 97 reflections intensity decay: none

#### Refinement

*R*[*F* ^2^ > 2σ(*F* ^2^)] = 0.056
*wR*(*F* ^2^) = 0.162
*S* = 1.03
2824 reflections
225 parameters
12 restraints
H atoms treated by a mixture of independent and constrained refinement
Δρ_max_ = 1.01 e Å^−3^
Δρ_min_ = −0.66 e Å^−3^

### 

Data collection: *XSCANS* (Siemens, 1996 ▶); cell refinement: *XSCANS*; data reduction: *XSCANS*; program(s) used to solve structure: *SHELXS97* (Sheldrick, 2008 ▶); program(s) used to refine structure: *SHELXL97* (Sheldrick, 2008 ▶); molecular graphics: *XP* in *SHELXTL* (Sheldrick, 2008 ▶); software used to prepare material for publication: *SHELXL97*.

## Supplementary Material

Crystal structure: contains datablocks global, I. DOI: 10.1107/S1600536808038154/bg2219sup1.cif

Structure factors: contains datablocks I. DOI: 10.1107/S1600536808038154/bg2219Isup2.hkl

Additional supplementary materials:  crystallographic information; 3D view; checkCIF report

## Figures and Tables

**Table 1:** Hydrogen-bond geometry (Å, °)

*D*—H⋯*A*	*D*—H	H⋯*A*	*D*⋯*A*	*D*—H⋯*A*
O1—H1*W*2⋯O2	0.85 (4)	2.04 (5)	2.885 (9)	176 (9)
O1—H1*W*1⋯Cl1	0.83 (4)	2.30 (4)	3.127 (7)	180 (9)
O2—H2*W*1⋯Cl1^i^	0.84 (4)	2.48 (4)	3.317 (7)	175 (6)
O2—H2*W*2⋯Cl2	0.84 (3)	2.33 (3)	3.165 (5)	173 (7)
O3—H3*W*1⋯Cl1^ii^	0.85 (6)	2.33 (6)	3.161 (5)	166 (6)
O3—H3*W*2⋯Cl2^iii^	0.84 (4)	2.27 (5)	3.095 (5)	170 (7)
O4—H4*W*1⋯O1	0.79 (5)	2.06 (5)	2.841 (8)	169 (5)
O4—H4*W*2⋯O3	0.81 (3)	2.00 (3)	2.795 (8)	168 (7)

Symmetry codes: (i) 
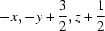
; (ii) 
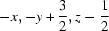
; (iii) 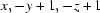; (iv) 
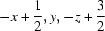
; (v) 
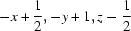
.

**Fig. 1.**
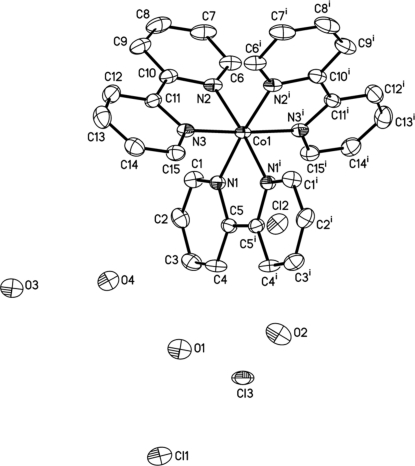
ORTEP view of the title compound. The dispalcement ellipsoids are drawn at 40% probability level. [Symmetry code: (i) -x + 1/2, y, -z + 3/2]

**Fig. 2.**
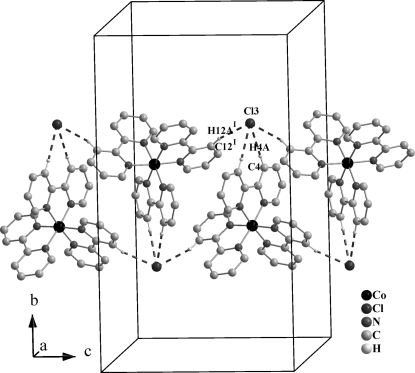
The one-dimensional chain of the [Co(phen)3]3+ cations along with [001] direction.

**Fig. 3.**
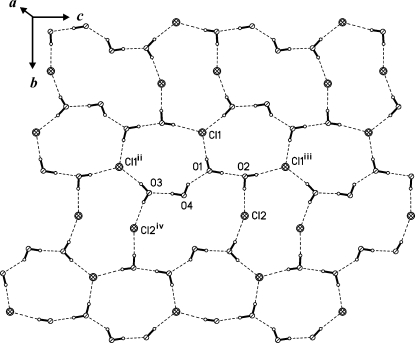
The two-dimensional water-chloride framework parallel to (100). [Symmetry codes: (ii) -x, -y + 3/2, z - 1/2; (iii) -x, -y + 3/2, z + 1/2; (iv): x, -y + 1, -z + 1]

**Fig. 4.**
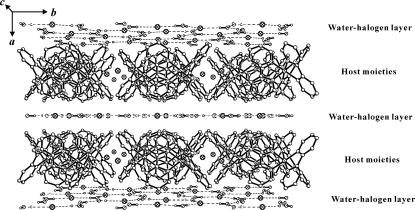
Packing diagram of the supramolecular edifice viewed along the crystallographic c axis.

## supplementary crystallographic information

### Comment

Due to the central role that water plays in biological and chemical processes, research on its structure, properties and functions has attracted the scientist's attention (Liu, *et al.*, 1996; Nauta & Miller, 2000; Ludwig, 2001; Mir & Vittal, 2007). However, there are only a few reports focused on the experimental identificaion and analysis of hydrogen-bond networks between water of crystallization and chloride counterions in crystalline materials. As a few examples we can mention [*cis*-α-(trine)CoCl_2_]Cl.3H_2_O (Saha & Bernal, 2005) where a two-dimensional layered structure containing [Cl_2_(H_2_O)_4_] six-membered rings build up. In the crystal structure of {*p*-C_6_H_4_[CH_2_OCH_2_C(pz)_3_]_2_[Ru(*p*-cymene)]_2_}Cl_4_.14H_2_O two-dimensional layers built from four-membered [Cl(H_2_O)_3_], five-membered [Cl(H_2_O)_4_], six-membered [Cl(H_2_O)_5_] and seven-membered [Cl_3_(H_2_O)_4_] rings formed through hydrogen bond self-assembly (Reger, *et al.*, 2006). A hybrid water-chloride structure of 14 water molecules and 4 Cl^-^ anions connected into layers is formed in the crystal structure of an europium complex [Eu_3_(BDC)_3_(phen)_3_Cl(H_2_O)_6_]Cl_2_.4H_2_O (Li, *et al.*, 2007). The references suggest that a great variety of mixed water-chloride supramolecular self-assemblies is possible. Herein, we describe a structure of a cobalt(III) complex containing discrete tris(2,2'-bipyridine)cobalt(III) cations and infinite layers of water and chloride anions, and where seven-membered (Cl_2_(H_2_O)_5_), eight-membered (Cl_4_(H_2_O)_4_) and ten-membered (Cl_2_(H_2_O)_8_) rings can be found.

The crystal structure of the title compound is composed of [Co(bpy)_3_]^3+^ complex cations, Cl^-^ anions and crystal H_2_O molecules (Fig. 1). Within the trivalent complex cations, the Co atoms are each surrounded by six N atoms of three chelating bpy ligands to complete a distorted octahedral coordination with d(Co—N) = 1,928 (3)–1.939 (3) Å, the *cis* and *trans* N—Co—N bond angles in the range 83.50 (19)–93.82 (14) and 175.23 (14)–176.38 (19)°, respectively. Such distances are similar to those found in other related structures (Hernández-Molina, *et al.*, 1998). The complex cation exhibits *C*_2_ symmetry with the twofold axis through the central Co atom and bisecting the C5—C5^i^ bond of one bpy ligand (i = -*x* + 1/2, *y*, -*z* + 3/2), as well as one of the Cl^-^ anions (Cl3). Through a C—H···Cl weak hydrogen bond the [Co(phen)_3_]^3+^ moieties are interlink into one-dimensional chains along the [001] direction (Fig.2). There are no π-π stacking interactions in the chains.

The most starling feature of the solid-state structure of 1 is the hydrogen-bonding interactions of the four water molecules and the remaining two Cl^-^ anions (Cl1 and Cl2), which lay on a mirror plane. The four crystal water molecules (O1, O2, O3,and O4) locate at the crystallographic 8f position and through intermolecular hydrogen bonds determine a linear water tetramer (H_2_O)_4_. The O···O distances span the range 2.795 (8)–2.885 (9) Å. Interestingly, such tetrameric water groups are further hydrogen-bond-interacting with the Cl^-^ anions to complete a two-dimensional water-chloride framework parallel to (100). As Shown in Fig. 3, the two-dimensional layers are composed by seven- (five water molecules and two Cl^-^ anions), eight- (four water molecules and four Cl^-^ anions) and ten-membered (eight water molecules and two Cl^-^ anions) rings through O—H···O and O—H···Cl interactions (Table 1). The O···Cl distances range from 3.095 (5) to 3.317 (7) Å and O—H···Cl angles span the range 166 (6) to 180 (9)°. The water-chloride layer can be viewed as building blocks, which are pillared by [Co(bpy)_3_]^3+^ spacers to produce an infinite three-dimensional supramolecular edifice. In this sense, the layer is different from already reported water clusters and other morphologies, which are situated within the host cavities or channels generated by organic and inorganic moieties (Fig. 4).

### Experimental

Addition of 10 ml CH_3_OH containing 0.162 g (1.04 mmol) 2,2'-bipyridine (bpy) to an aqueous solution of 0.238 g (1.00 mmol) CoCl_2_.6H_2_O in 10 ml H_2_O gave a yellow solution, then dropwise added 3 ml 30% H_2_O_2_ solution to the mixture, stirred for *ca* half an hour. The resulting light-yellow solution (PH = 6.48) was allowed to stand at room temperature. After 8 days, a small amount of yellow block crystals had grown.

### Refinement

H atoms bonded to C atoms were palced in geometrically calculated position and were refined using a riding model, with *U*_iso_(H) = 1.2 *U*_eq_(C). H atoms attached to O atoms were found in a difference Fourier synthesis and were refined using a riding model, with the O—H distances fixed as initially found and with *U*_iso_(H) values set at 1.5 *U*eq(O).

### Crystal data

**Table:**

[Co(C_10_H_8_N_2_)_3_]Cl_3_·4H_2_O	*F*_000_ = 2912
*M**_r_* = 705.90	*D*_x_ = 1.507 Mg m^−^^3^
Orthorhombic, *C**m**c**a*	Mo *K*α radiation λ = 0.71073 Å
Hall symbol: -C 2bc 2	Cell parameters from 25 reflections
*a* = 20.171 (4) Å	θ = 5.0–12.5º
*b* = 23.170 (5) Å	µ = 0.86 mm^−^^1^
*c* = 13.316 (3) Å	*T* = 295 (2) K
*V* = 6223 (2) Å^3^	Block, yellow
*Z* = 8	0.12 × 0.10 × 0.08 mm

### Data collection

**Table:**

Bruker P4 diffractometer	*R*_int_ = 0.049
Radiation source: fine-focus sealed tube	θ_max_ = 25.0º
Monochromator: graphite	θ_min_ = 1.8º
*T* = 295(2) K	*h* = −23→1
θ/2θ scans	*k* = −1→27
Absorption correction: ψ scan(XSCANS; Siemens, 1996)	*l* = −1→15
*T*_min_ = 0.905, *T*_max_ = 0.929	3 standard reflections
3430 measured reflections	every 97 reflections
2824 independent reflections	intensity decay: none
1980 reflections with *I* > 2σ(*I*)	

### Refinement

**Table:**

Refinement on *F*^2^	Secondary atom site location: difference Fourier map
Least-squares matrix: full	Hydrogen site location: inferred from neighbouring sites
*R*[*F*^2^ > 2σ(*F*^2^)] = 0.056	H atoms treated by a mixture of independent and constrained refinement
*wR*(*F*^2^) = 0.162	*w* = 1/[σ^2^(*F*_o_^2^) + (0.1023*P*)^2^] where *P* = (*F*_o_^2^ + 2*F*_c_^2^)/3
*S* = 1.03	(Δ/σ)_max_ < 0.001
2824 reflections	Δρ_max_ = 1.01 e Å^−^^3^
225 parameters	Δρ_min_ = −0.66 e Å^−^^3^
12 restraints	Extinction correction: none
Primary atom site location: structure-invariant direct methods	

### Special details

**Table:**

Geometry. All e.s.d.'s (except the e.s.d. in the dihedral angle between two l.s. planes) are estimated using the full covariance matrix. The cell e.s.d.'s are taken into account individually in the estimation of e.s.d.'s in distances, angles and torsion angles; correlations between e.s.d.'s in cell parameters are only used when they are defined by crystal symmetry. An approximate (isotropic) treatment of cell e.s.d.'s is used for estimating e.s.d.'s involving l.s. planes.
Refinement. Refinement of *F*^2^ against ALL reflections. The weighted *R*-factor *wR* and goodness of fit *S* are based on *F*^2^, conventional *R*-factors *R* are based on *F*, with *F* set to zero for negative *F*^2^. The threshold expression of *F*^2^ > σ(*F*^2^) is used only for calculating *R*-factors(gt) *etc*. and is not relevant to the choice of reflections for refinement. *R*-factors based on *F*^2^ are statistically about twice as large as those based on *F*, and *R*- factors based on ALL data will be even larger.

### Fractional atomic coordinates and isotropic or equivalent isotropic displacement parameters (Å^2^)

**Table:**

	*x*	*y*	*z*	*U*_iso_*/*U*_eq_	
Co1	0.2500	0.40805 (3)	0.7500	0.0230 (2)	
Cl1	0.0000	0.80958 (8)	0.57842 (16)	0.0580 (5)	
Cl2	0.0000	0.52160 (8)	0.83302 (13)	0.0487 (5)	
Cl3	0.2500	0.71245 (7)	0.7500	0.0508 (5)	
N1	0.19627 (15)	0.47048 (14)	0.6973 (2)	0.0254 (7)	
N2	0.19583 (15)	0.34934 (14)	0.6871 (3)	0.0278 (8)	
N3	0.29820 (15)	0.40542 (14)	0.6251 (2)	0.0267 (8)	
C1	0.13885 (18)	0.46643 (19)	0.6478 (3)	0.0328 (10)	
H1A	0.1227	0.4300	0.6316	0.039*	
C2	0.1025 (2)	0.51433 (19)	0.6198 (3)	0.0367 (11)	
H2A	0.0628	0.5100	0.5852	0.044*	
C3	0.1256 (2)	0.5687 (2)	0.6436 (3)	0.0382 (11)	
H3A	0.1016	0.6014	0.6257	0.046*	
C4	0.1849 (2)	0.57363 (18)	0.6944 (3)	0.0370 (11)	
H4A	0.2019	0.6098	0.7102	0.044*	
C5	0.21896 (19)	0.52388 (17)	0.7216 (3)	0.0267 (9)	
C6	0.1430 (2)	0.32196 (18)	0.7267 (4)	0.0368 (11)	
H6A	0.1316	0.3286	0.7934	0.044*	
C7	0.1052 (2)	0.28387 (19)	0.6695 (4)	0.0453 (12)	
H7A	0.0695	0.2646	0.6983	0.054*	
C8	0.1204 (2)	0.27501 (19)	0.5722 (4)	0.0486 (13)	
H8A	0.0939	0.2512	0.5330	0.058*	
C9	0.1750 (2)	0.30103 (18)	0.5309 (4)	0.0407 (11)	
H9A	0.1867	0.2940	0.4645	0.049*	
C10	0.2128 (2)	0.33844 (17)	0.5904 (3)	0.0300 (9)	
C11	0.27255 (19)	0.36858 (17)	0.5557 (3)	0.0284 (9)	
C12	0.3015 (2)	0.3604 (2)	0.4646 (3)	0.0410 (11)	
H12A	0.2824	0.3358	0.4176	0.049*	
C13	0.3601 (2)	0.3892 (2)	0.4427 (4)	0.0452 (12)	
H13A	0.3813	0.3835	0.3815	0.054*	
C14	0.3861 (2)	0.4261 (2)	0.5121 (3)	0.0397 (11)	
H14A	0.4251	0.4459	0.4984	0.048*	
C15	0.3545 (2)	0.43360 (18)	0.6017 (3)	0.0329 (10)	
H15A	0.3725	0.4591	0.6482	0.039*	
O1	0.0000	0.6769 (3)	0.6215 (5)	0.092 (2)	
H1W1	0.0000	0.7122 (17)	0.610 (8)	0.138*	
H1W2	0.0000	0.673 (5)	0.685 (3)	0.138*	
O2	0.0000	0.6582 (2)	0.8357 (5)	0.0643 (15)	
H2W1	0.0000	0.664 (3)	0.898 (3)	0.096*	
H2W2	0.0000	0.6220 (14)	0.829 (6)	0.096*	
O3	0.0000	0.6013 (2)	0.2581 (4)	0.0701 (17)	
H3W1	0.0000	0.620 (3)	0.203 (4)	0.105*	
H3W2	0.0000	0.5664 (14)	0.241 (6)	0.105*	
O4	0.0000	0.5921 (2)	0.4674 (4)	0.0566 (13)	
H4W1	0.0000	0.619 (2)	0.504 (4)	0.085*	
H4W2	0.0000	0.600 (3)	0.408 (2)	0.085*	

### Atomic displacement parameters (Å^2^)

**Table:**

	*U*^11^	*U*^22^	*U*^33^	*U*^12^	*U*^13^	*U*^23^
Co1	0.0243 (4)	0.0150 (4)	0.0297 (4)	0.000	−0.0016 (3)	0.000
Cl1	0.0727 (12)	0.0362 (10)	0.0651 (12)	0.000	0.000	−0.0075 (9)
Cl2	0.0512 (9)	0.0463 (10)	0.0486 (10)	0.000	0.000	−0.0087 (8)
Cl3	0.0791 (12)	0.0197 (8)	0.0537 (10)	0.000	0.0244 (9)	0.000
N1	0.0284 (17)	0.0208 (17)	0.0269 (17)	−0.0004 (14)	0.0009 (14)	0.0003 (15)
N2	0.0262 (17)	0.0184 (17)	0.039 (2)	−0.0013 (14)	−0.0021 (14)	0.0005 (15)
N3	0.0277 (17)	0.0194 (16)	0.0331 (18)	0.0035 (14)	0.0010 (14)	0.0009 (15)
C1	0.026 (2)	0.030 (2)	0.042 (2)	0.0006 (18)	−0.0036 (19)	−0.002 (2)
C2	0.031 (2)	0.043 (3)	0.037 (2)	0.0062 (19)	−0.0053 (19)	0.007 (2)
C3	0.037 (2)	0.032 (2)	0.046 (3)	0.0104 (19)	−0.002 (2)	0.007 (2)
C4	0.043 (3)	0.016 (2)	0.052 (3)	0.0019 (18)	0.000 (2)	0.003 (2)
C5	0.0297 (19)	0.022 (2)	0.028 (2)	−0.0002 (18)	0.0029 (17)	0.0022 (17)
C6	0.034 (2)	0.022 (2)	0.054 (3)	−0.0020 (19)	−0.001 (2)	0.001 (2)
C7	0.034 (2)	0.026 (2)	0.076 (4)	−0.0095 (19)	−0.005 (2)	−0.002 (2)
C8	0.045 (3)	0.029 (3)	0.072 (4)	−0.005 (2)	−0.018 (3)	−0.015 (3)
C9	0.044 (3)	0.028 (2)	0.051 (3)	0.000 (2)	−0.008 (2)	−0.012 (2)
C10	0.031 (2)	0.022 (2)	0.038 (2)	0.0031 (17)	−0.0054 (19)	−0.0041 (19)
C11	0.034 (2)	0.021 (2)	0.030 (2)	0.0027 (17)	−0.0067 (18)	−0.0040 (18)
C12	0.049 (3)	0.037 (2)	0.037 (3)	0.007 (2)	−0.005 (2)	−0.006 (2)
C13	0.044 (3)	0.056 (3)	0.036 (3)	0.014 (2)	0.008 (2)	0.003 (2)
C14	0.038 (2)	0.037 (2)	0.044 (3)	0.000 (2)	0.006 (2)	0.007 (2)
C15	0.033 (2)	0.027 (2)	0.040 (3)	−0.0023 (18)	0.0043 (19)	0.000 (2)
O1	0.168 (7)	0.043 (3)	0.064 (4)	0.000	0.000	0.000 (3)
O2	0.069 (3)	0.053 (3)	0.071 (4)	0.000	0.000	0.016 (3)
O3	0.112 (5)	0.038 (3)	0.060 (4)	0.000	0.000	0.004 (3)
O4	0.078 (3)	0.043 (3)	0.048 (3)	0.000	0.000	−0.006 (3)

### Geometric parameters (Å, °)

**Table:**

Co1—N3^i^	1.928 (3)	C7—C8	1.347 (7)
Co1—N3	1.928 (3)	C7—H7A	0.9300
Co1—N2^i^	1.935 (3)	C8—C9	1.370 (7)
Co1—N2	1.935 (3)	C8—H8A	0.9300
Co1—N1	1.939 (3)	C9—C10	1.401 (6)
Co1—N1^i^	1.939 (3)	C9—H9A	0.9300
N1—C1	1.336 (5)	C10—C11	1.467 (6)
N1—C5	1.358 (5)	C11—C12	1.360 (6)
N2—C6	1.348 (5)	C12—C13	1.388 (7)
N2—C10	1.355 (5)	C12—H12A	0.9300
N3—C15	1.347 (5)	C13—C14	1.363 (7)
N3—C11	1.360 (5)	C13—H13A	0.9300
C1—C2	1.381 (6)	C14—C15	1.363 (6)
C1—H1A	0.9300	C14—H14A	0.9300
C2—C3	1.379 (6)	C15—H15A	0.9300
C2—H2A	0.9300	O1—H1W1	0.83 (3)
C3—C4	1.379 (6)	O1—H1W2	0.85 (3)
C3—H3A	0.9300	O2—H2W1	0.85 (3)
C4—C5	1.390 (6)	O2—H2W2	0.84 (3)
C4—H4A	0.9300	O3—H3W1	0.85 (3)
C5—C5^i^	1.462 (8)	O3—H3W2	0.84 (3)
C6—C7	1.394 (6)	O4—H4W1	0.80 (3)
C6—H6A	0.9300	O4—H4W2	0.81 (3)
			
N3^i^—Co1—N3	176.38 (19)	N1—C5—C5^i^	114.3 (2)
N3^i^—Co1—N2^i^	83.63 (14)	C4—C5—C5^i^	123.9 (3)
N3—Co1—N2^i^	93.82 (14)	N2—C6—C7	121.1 (4)
N3^i^—Co1—N2	93.82 (14)	N2—C6—H6A	119.4
N3—Co1—N2	83.63 (14)	C7—C6—H6A	119.4
N2^i^—Co1—N2	90.69 (19)	C8—C7—C6	119.8 (4)
N3^i^—Co1—N1	93.09 (13)	C8—C7—H7A	120.1
N3—Co1—N1	89.61 (13)	C6—C7—H7A	120.1
N2^i^—Co1—N1	175.23 (14)	C7—C8—C9	120.2 (4)
N2—Co1—N1	92.99 (13)	C7—C8—H8A	119.9
N3^i^—Co1—N1^i^	89.61 (13)	C9—C8—H8A	119.9
N3—Co1—N1^i^	93.09 (13)	C8—C9—C10	118.9 (5)
N2^i^—Co1—N1^i^	92.99 (13)	C8—C9—H9A	120.6
N2—Co1—N1^i^	175.23 (14)	C10—C9—H9A	120.6
N1—Co1—N1^i^	83.50 (19)	N2—C10—C9	121.0 (4)
C1—N1—C5	118.3 (3)	N2—C10—C11	114.7 (3)
C1—N1—Co1	127.6 (3)	C9—C10—C11	124.3 (4)
C5—N1—Co1	113.9 (2)	N3—C11—C12	122.0 (4)
C6—N2—C10	119.0 (4)	N3—C11—C10	113.4 (3)
C6—N2—Co1	127.4 (3)	C12—C11—C10	124.6 (4)
C10—N2—Co1	113.5 (3)	C11—C12—C13	119.1 (4)
C15—N3—C11	117.9 (4)	C11—C12—H12A	120.5
C15—N3—Co1	127.6 (3)	C13—C12—H12A	120.5
C11—N3—Co1	114.5 (3)	C14—C13—C12	119.1 (4)
N1—C1—C2	122.5 (4)	C14—C13—H13A	120.5
N1—C1—H1A	118.8	C12—C13—H13A	120.5
C2—C1—H1A	118.8	C13—C14—C15	119.6 (4)
C3—C2—C1	119.5 (4)	C13—C14—H14A	120.2
C3—C2—H2A	120.3	C15—C14—H14A	120.2
C1—C2—H2A	120.3	N3—C15—C14	122.3 (4)
C2—C3—C4	118.8 (4)	N3—C15—H15A	118.9
C2—C3—H3A	120.6	C14—C15—H15A	118.9
C4—C3—H3A	120.6	H1W1—O1—H1W2	107 (7)
C3—C4—C5	119.2 (4)	H2W1—O2—H2W2	106 (5)
C3—C4—H4A	120.4	H3W1—O3—H3W2	106 (5)
C5—C4—H4A	120.4	H4W1—O4—H4W2	114 (5)
N1—C5—C4	121.8 (4)		

Symmetry codes: (i) −*x*+1/2, *y*, −*z*+3/2.

### Hydrogen-bond geometry (Å, °)

**Table:**

*D*—H···*A*	*D*—H	H···*A*	*D*···*A*	*D*—H···*A*
O1—H1W2···O2	0.85 (4)	2.04 (5)	2.885 (9)	176 (9)
O1—H1W1···Cl1	0.83 (4)	2.30 (4)	3.127 (7)	180 (9)
O2—H2W1···Cl1^ii^	0.84 (4)	2.48 (4)	3.317 (7)	175 (6)
O2—H2W2···Cl2	0.84 (3)	2.33 (3)	3.165 (5)	173 (7)
O3—H3W1···Cl1^iii^	0.85 (6)	2.33 (6)	3.161 (5)	166 (6)
O3—H3W2···Cl2^iv^	0.84 (4)	2.27 (5)	3.095 (5)	170 (7)
O4—H4W1···O1	0.79 (5)	2.06 (5)	2.841 (8)	169 (5)
O4—H4W2···O3	0.81 (3)	2.00 (3)	2.795 (8)	168 (7)
C1—H1A···N2	0.93	2.49	2.993 (5)	114
C4—H4A···Cl3	0.93	2.62	3.552 (4)	179
C6—H6A···N3^i^	0.93	2.52	3.007 (6)	113
C8—H8A···Cl1^iv^	0.93	2.79	3.710 (5)	172
C12—H12A···Cl3^v^	0.93	2.58	3.477 (4)	162
C14—H14A···Cl2^v^	0.93	2.77	3.526 (4)	139
C15—H15A···N1^i^	0.93	2.49	2.991 (5)	114

Symmetry codes:  (ii) −*x*, −*y*+3/2, *z*+1/2; (iii) −*x*, −*y*+3/2, *z*−1/2; (iv) *x*, −*y*+1, −*z*+1; (i) −*x*+1/2, *y*, −*z*+3/2; (v) −*x*+1/2, −*y*+1, *z*−1/2.
